# Predictive Factors for Peritoneal Metastases in Colorectal Cancer: A Retrospective Study Conducted at a Tertiary Care Center in Riyadh, Saudi Arabia

**DOI:** 10.7759/cureus.77150

**Published:** 2025-01-08

**Authors:** Ibrahim S AlSamaani, Nayef Alzahrani, Fahad Aljehaiman, Ghadah B Alghbewi, Nejood Alsheikh, Alaa S Aljulayfi, Abdullah M Alsubaie, Aymen S Alrasheed, Ramesh Vishwakarma

**Affiliations:** 1 General Surgery, King Abdulaziz Medical City Riyadh, Riyadh, SAU; 2 Research, King Abdullah International Medical Research Center (KAIMRC), Riyadh, SAU; 3 Surgical Oncology, King Abdulaziz Medical City Riyadh, Riyadh, SAU; 4 Medicine, King Saud Bin Abdulaziz University for Health Sciences, Riyadh, SAU; 5 Internal Medicine, King Saud Bin Abdulaziz University for Health Sciences, Riyadh, SAU; 6 Internal Medicine, King Abdulaziz Medical City Riyadh, Riyadh, SAU; 7 Statistics, University of East Anglia, Norwich, GBR

**Keywords:** colorectal cancer, cytoreductive surgery (crs), hyperthermic intraperitoneal chemotherapy (hipec), ovarian metastasis, peritoneal metastasis

## Abstract

Background

The incidence of colorectal cancer (CRC) is increasing nationally, emphasizing the importance of early detection. Late-stage diagnosis, particularly with peritoneal metastasis (PM), is associated with poor outcomes despite advancements in treatment. PM poses a challenge owing to the lack of standardized treatment, although cytoreductive surgery (CRS) with hyperthermic intraperitoneal chemotherapy (HIPEC) shows promise in improving survival rates. This study aimed to document the incidence and risk factors for patients presenting with PM following CRC.

Materials and methods

This retrospective chart review study was conducted at King Abdulaziz Medical City in Saudi Arabia. Patients of all ages and genders who underwent surgical resection for CRC from January 2016 to December 2021 were included. Patients with PM at primary index surgery, distant organ metastasis to the lung or liver, or other types of cancer were excluded. The data was analyzed via SPSS Statistics version 27.0 (IBM Corp. Released 2020. IBM SPSS Statistics for Windows, Version 27.0. Armonk, NY: IBM Corp.), t-test, Wilcoxon rank-sum test, chi-square test, and Fisher’s exact tests.

Results

Among 565 patients with CRC, 65 patients (11.5%) developed PM. Tumor characteristics such as T4 stage, mucinous tumors, elevated CEA, and the presence of ovarian metastasis at primary resection were independent factors for peritoneal recurrence. However, in univariate and multivariate associations, the presence of ovarian metastasis, mucinous tumors, postoperative CEA levels exceeding 5 ng/ml, and perforation are significant risk factors for PM development post-surgery, with hazard ratios of 6.457 (CI 2.481-16.801) and 4.057 (2.029-8.115), respectively.

Conclusion

Mucinous tumors and ovarian metastasis are significant risk factors for PM development, emphasizing the importance of early identification for tailored treatment strategies such as CRS and HIPEC.

## Introduction

Colorectal cancer (CRC) is a leading cause of cancer-related morbidity and mortality worldwide. Advances in detection, screening, referral pathways, centralized care, improved surgical techniques, systemic chemotherapy, and the introduction of biological agents have significantly improved survival rates following diagnosis [1]. In Saudi Arabia, the one-, three-, and five-year survival rates for CRC patients are reported at 83.09%, 65%, and 52%, respectively [2]. Despite these advancements, the prognosis for patients with peritoneal metastasis (PM) of CRC remains poor, highlighting the need for focused research and innovative treatment approaches [3].

PM occurs when CRC spreads to the peritoneal cavity, a condition associated with significant morbidity and limited treatment options. Currently, there is no universally established standard treatment for PM. Traditional approaches, such as systemic chemotherapy or surgery alone, have shown minimal impact on improving patient survival. In response to this challenge, Sugarbaker and Yonemura introduced a multimodal treatment approach involving cytoreductive surgery (CRS) combined with hyperthermic intraperitoneal chemotherapy (HIPEC). This technique involves the surgical removal of visible tumors followed by the application of heated chemotherapy to target residual microscopic peritoneal disease. Studies have demonstrated that achieving complete cytoreduction is associated with improved survival outcomes, emphasizing the potential of CRS-HIPEC as an effective treatment modality [4].

Despite the growing body of evidence supporting CRS-HIPEC, data on CRC with PM remains sparse in Saudi Arabia. The epidemiology, management practices, and outcomes of patients with CRC and PM in the region are poorly understood due to the lack of published reports. Addressing this knowledge gap is crucial for developing tailored strategies to improve patient care.

The primary objective of this study was to evaluate the incidence and demographic characteristics of patients with PM following primary CRC resection at a tertiary care center in Saudi Arabia. The secondary objectives were to identify predictive factors for PM in CRC patients, including clinical presentation, tumor characteristics and location, postoperative carcinoembryonic antigen (CEA) levels, ovarian metastasis, and the role of adjuvant chemotherapy. Our findings aim to contribute to a better understanding of CRC with PM in the region and provide valuable insights for clinicians and policymakers working to optimize care for these patients.

## Materials and methods

This study was conducted at King Abdulaziz Medical City (KAMC) in Riyadh, a tertiary care facility under the National Guard Health Affairs, established in May 1983, with an approximate capacity of 3,133 beds. It employed a retrospective cohort design, using non-probability consecutive sampling to include all patients meeting the inclusion criteria. The study population comprised male and female patients of all ages who underwent primary surgical resection for CRC at KAMC between January 2016 and December 2021.

Ethical approval for the study was granted in July 2023 by the Institutional Review Board of King Abdullah International Medical Research Center, with approval number IRB/1798/23. Since the study was retrospective, informed consent was not required. To ensure confidentiality and privacy, no patient-identifiable information, such as names, medical record numbers (MRNs), or ID numbers, was collected. Instead, MRNs were replaced with unique serial numbers. All collected data were securely stored, with hard and soft copies maintained within the National Guard Health Affairs premises.

Data were obtained through a comprehensive chart review using the BESTcare system (Saudi-Korean Health Informatics Company, Riyadh, Saudi Arabia). Only members of the research team were responsible for data collection. The inclusion criteria encompassed all patients who underwent primary surgical resection for CRC within the study period. Patients were excluded if they had a prior diagnosis of malignancies other than CRC (e.g., liver or lung cancer), had PM identified either radiologically (via CT or PET scans) or during surgery at the time of primary CRC resection, or if they did not undergo surgical intervention.

The collected data encompassed a range of clinical, pathological, and demographic variables. Patient demographics included age and gender. The clinical presentation of the primary cancer was categorized into bleeding, obstruction, perforation, or other presentation. Information on the tumor site was also documented, including rectosigmoid, rectum, right colon, left colon, and transverse colon. Key predictive factors analyzed included tumor stage, type of primary pathology, surgical margins, and tumor differentiation. CEA levels were measured, and data on the administration of adjuvant chemotherapy were recorded. Additionally, the presence of ovarian metastasis was also collected to be assessed as a possible predictive factor.

A total of 565 patients met the inclusion criteria. The cohort was divided into two groups for analysis: patients who developed PM after primary CRC surgery and those who did not. Statistical analyses were performed using SPSS Statistics version 27.0 (IBM Corp. Released 2020. IBM SPSS Statistics for Windows, Version 27.0. Armonk, NY: IBM Corp.). Categorical variables were analyzed using the chi-square test or Fisher’s exact test as appropriate. For continuous variables, comparisons were made using the Student’s t-test for normally distributed data or the Mann-Whitney U test for non-normally distributed data. The normality of continuous variables was assessed using the Shapiro-Wilk test and graphical tools such as histograms and Q-Q plots.

To identify factors associated with outcomes, univariate and multivariate logistic regression analyses were conducted. Odds ratios (ORs) with 95% confidence intervals (CIs) were reported to quantify associations. The goodness-of-fit of the logistic regression model was evaluated using the Hosmer-Lemeshow goodness-of-fit test. A p-value of <0.05 was considered statistically significant for all analyses.

## Results

Patient demographics and clinical presentations are summarized in Table 1. A total of 565 patients were included in this study, comprising 347 males (61.4%) and 218 females (38.5%). Among these, 65 patients (11.5%) developed PM after primary CRC resection, with 34 males (52.3%) and 31 females (47.7%) affected. The median age of patients who developed PM was 62 years. The clinical presentation in this group was dominated by obstruction, observed in 36 patients (55.4%), followed by bleeding in 17 patients (26.2%) and perforation in one patient (1.5%). The primary tumor was most frequently located in the rectosigmoid region, accounting for 22 cases (33.8%) in the PM group.

**Table 1:** Patients' presentation Q1: first Quartiles, Q3: third quartiles, PM: peritoneal metastasis * t-test, ^ Wilcoxon rank-sum test is used to calculate the p-value, ^^ chi-square test is used to calculate the p-value, ** Fisher's exact test is used to calculate the p-value

Variables	Colorectal patients with no PM N=500	Colorectal patients with recurrent PM N=65	p-value
Age (years), median (Q1, Q3)	65 (56.0, 73.0)	62 (54.0, 71.0)	0.0963^
Gender			
Female	187 (37.4)	31 (47.7)	0.1088^^
Male	313 (62.6)	34 (52.3)	
Presentation of primary cancer			
Bleeding	218 (43.6)	17 (26.2)	0.0505**
Obstruction	224 (44.8)	36 (55.4)	
Perforation	4 (0.8)	1 ( 1.5)	
Others	54 (10.8)	11 (16.9)	
Site of primary cancer			
Rectosigmoid	147 (29.4)	22 (33.8)	0.1132^^
Rectum	138 (27.6)	9 (13.8)	
Right colon	104 (20.8)	15 (23.1)	
Left colon	98 (19.6)	15 (23.1)	
Transverse colon	13 ( 2.6)	4 (6.2)	

Table 2 highlights the tumor characteristics and predictive factors for PM. The distribution of patients across all tumor stages was significantly different between the two groups (p < 0.001). Among patients who developed PM, T3 and T4 stages were the most common, observed in 27 (41.5%) and 30 patients (46.2%), respectively, while early stages T1 and T2 were less common, with only two (3.1%) and one (1.5%) cases, respectively. The type of primary pathology also demonstrated a statistically significant difference between groups (p = 0.0314). Adenocarcinoma was the most common pathology in the PM group, present in 83.1% of cases (p = 0.0314), followed by mucinous carcinoma in 13.8% (p = 0.0314) and signet cells in 3.1% (p = 0.0920). Positive surgical margins were more frequent among patients with PM (10.8%) compared to those without PM (5.6%), although the difference was not statistically significant (p = 0.1039). Moderate differentiation was the predominant histopathological finding in the PM group, observed in 59 patients (90.8%), but tumor differentiation did not show a significant difference between the two groups (p = 0.2356). CEA levels greater than 5 ng/mL were significantly different between the two groups, observed in 33.8% of patients in the PM group compared to 17.8% in the non-PM group (p = 0.0028). Adjuvant chemotherapy was administered to 54 patients with PM (83.1%), which was significantly different compared to patients without PM (p < 0.0001). Lastly, ovarian metastasis was significantly associated with PM, occurring in 11 patients (16.9%) in the PM group compared to only 1.6% in the non-PM group (p < 0.0001).

**Table 2:** Predictive factors CEA: carcinoembryonic antigen * t-test, ^ Wilcoxon rank-sum test is used to calculate the p-value, ^^ chi-square test is used to calculate the p-value, ** Fisher's exact test is used to calculate the p-value

Predictive factors	Variables	Colorectal patients with no peritoneal mets N=500	Colorectal patients with recurrent peritoneal mets N=65	p-value
Tumor stage	T1	8 (1.6)	2 (3.1)	<000.1^^
	T2	61 (12.2)	1 (1.5)	
	T3	311 (62.2)	27 (41.5)	
	T4	106 (21.2)	30 (46.2)	
	Unknown	14 (2.8)	5 (7.6)	
Type of primary pathology	Adenocarcinoma	458 (91.6)	54 (83.1)	0.0314**
	Signet cells	4 ( 0.8)	2 (3.1)	0.0920**
	Mucinous	39 (7.8)	9 (13.8)	0.0314**
Margins	Positive	28 (5.6)	7 (10.8)	0.1039**
	Negative	472 (94.4)	58 (89.2)	
Differentiation	Well	20 (4.0)	1 (1.5)	0.2356**
	Moderately	437 (87.4)	59 (90.8)	
	Poorly	27 (5.4)	1 (1.5)	
	Undifferentiated	3 (0.6)	0 (0.0)	
	Unknown	13 (2.6)	4 (6.2)	
Postoperative CEA level	Less than 5 ng/ml	347 (69.4)	32 (49.2)	0.0028^^
	More than 5 ng/ml	89 (17.8)	22 (33.8)	
	Unknown	64 (12.8)	11 (16.9)	
Adjuvant chemotherapy	No	223 (44.6)	11 (16.9)	<0.0001^
	Yes	277 (55.4)	54 (83.1)	<0.0001^
Presence of ovarian metastasis	No	492 (98.4)	54 (83.1)	<0.0001**
	Yes	8 (1.6)	11 (16.9)	

The univariable and multivariable logistic regression analyses for predictive factors for PM in CRC patients are summarized in Table 3. Among clinical presentations, bleeding was associated with a reduced likelihood of PM in the univariable analysis (OR = 0.407, 95% CI: 0.183-0.904), but this association was not statistically significant in the multivariable model (OR = 0.576, 95% CI: 0.250-1.328). Neither obstruction nor perforation showed significant associations with PM in either univariable or multivariable analysis. The presence of mucinous tumors was significantly associated with PM in both univariable (OR = 2.909, 95% CI: 1.609-5.258) and multivariable analyses (OR = 2.472, 95% CI: 1.303-4.690). Ovarian metastasis emerged as the strongest predictor of PM, with highly significant associations in both univariable (OR = 11.890, 95% CI: 4.878-28.983) and multivariable models (OR = 6.457, 95% CI: 2.481-16.801). Adjuvant chemotherapy was also significantly associated with PM, with an increased likelihood observed in both univariable (OR = 4.199, 95% CI: 2.179-8.093) and multivariable analyses (OR = 4.057, 95% CI: 2.029-8.115). Regarding tumor staging, T2 staging was associated with a significantly reduced risk of PM in both univariable (OR = 0.083, 95% CI: 0.009-0.751) and multivariable analyses (OR = 0.073, 95% CI: 0.011-0.496). Similarly, T3 staging demonstrated a lower likelihood of PM in the multivariable model (OR = 0.264, 95% CI: 0.089-0.782). However, T4 staging was not significantly associated with PM in either analysis. Postoperative CEA levels greater than 5 ng/mL showed a positive association with PM in both univariable analysis (OR = 1.799, 95% CI: 0.818-3.953) and the multivariable model (OR = 1.824, 95% CI: 0.808-4.120), but this association was not statistically significant.

**Table 3:** Univariable and multivariable logistic regression analyses for predictive factors for PM in CRC patients PM: peritoneal metastasis, CI: confidence interval, OR: odds ratio, CEA: carcinoembryonic antigen, CRC: colorectal cancer

Predictive factors	Univariable logistic OR (95% CI)	Multivariable logistic OR (95% CI)
Bleeding	0.407 (0.183-0.904)	0.576 (0.250-1.328)
Obstruction	0.884 (0.429-1.819)	0.906 (0.431-1.906)
Perforation	1.292 (0.166-10.063)	1.104 (0.130-9.342)
Mucinous tumor	2.909 (1.609-5.258)	2.472 (1.303-4.690)
Ovarian metastasis	11.890 (4.878-28.983)	6.457 (2.481-16.801)
Adjuvant chemotherapy	4.199 (2.179-8.093)	4.057 (2.029-8.115)
Signet cells	3.952 (0.730-21.391)	2.246 (0.380-13.262)
T2 staging	0.083 (0.009-0.751)	0.073 (0.011-0.496)
T3 staging	0.347 (0.076-1.594)	0.264 (0.089-0.782)
T4 staging	0.801 (0.264-2.428)	0.761 (0.254-2.279)
Postoperative CEA levels less than 5 ng/ml	0.633 (0.301-1.330)	0.763 (0.355-1.638)
Postoperative CEA levels more than 5 ng/ml	1.799 (0.818-3.953)	1.824 (0.808-4.120)

## Discussion

CRC remains a significant health burden globally and is the leading cancer in men and the third most common cancer in women in Saudi Arabia, with increasing incidence rates [5]. While early-stage disease (stages I and II) offers a favorable prognosis, patients with advanced stages, particularly those with PM, face significantly worse outcomes [6-8]. This study provides a detailed analysis of PM incidence, demographics, and predictive factors in CRC patients, offering valuable insights into its clinical and pathological determinants.

Our findings provide a comprehensive analysis of univariate and multivariate associations between tumor characteristics and PM development. Factors such as a postoperative CEA level <5 ng/mL, T2 stage, T3 stage, and bleeding as the primary presentation were associated with a reduced risk of PM. Conversely, univariate analysis indicated that ovarian metastasis, adjuvant chemotherapy, mucinous tumors, postoperative CEA levels >5 ng/mL, and perforation were associated with an increased risk of PM. Obstruction, signet cells, and the T4 stage demonstrated unclear associations. Notably, the T3 stage was the most common tumor stage among patients without PM, whereas the T4 stage was most prevalent in patients with PM, which is consistent with the invasive nature of T4 tumors that involve adjacent organs or the visceral peritoneum [9,10]. A prior study similarly reported that the T4 stage was a significant predictor of metastasis in CRC patients with metachronous disease [11].

In the multivariate analysis, mucinous adenocarcinoma, ovarian metastasis, and adjuvant chemotherapy remained strongly associated with the development of PM. These findings suggest that these factors independently contribute to the risk of peritoneal dissemination. Perforation, while indicating a potential increased risk of PM, was not statistically significant due to the limited number of cases (only one patient in our dataset). Similarly, obstruction presented an uncertain risk. A meta-analysis published in 2019 reported both perforation and obstruction as significant predictors of decreased overall survival in patients undergoing CRS-HIPEC [12].

Our data also highlight the indeterminate role of signet cells in PM development, while mucinous tumors were strongly associated with an increased risk. This finding differs from a 2016 study that reported both signet cells and mucinous adenocarcinomas were associated with peritoneal dissemination compared with liver metastasis [13]. The difference may be attributed to the small sample size in our study, with only two cases of signet cells and nine cases of mucinous adenocarcinoma. Similarly, elevated postoperative CEA levels (>5 ng/mL) were significantly associated with PM in univariate analysis, while levels <5 ng/mL appeared protective. These findings align with a prior study that linked peritoneal effusion CEA levels >4 ng/mL to increased recurrence risk and lower survival rates [14].

Ovarian metastasis emerged as a significant independent predictor of PM, consistent with reports indicating that the peritoneum is the primary site of recurrence in ovarian cancer, with 75% of recurrences occurring intraperitoneally [15]. A 2010 study found that CRC patients with stages I-III ovarian metastases frequently developed PM and had poor survival outcomes [16]. Additionally, a French study in 2011 observed no signs of recurrence after second-look surgeries despite visible peritoneal carcinomatosis in 62% of patients with ovarian involvement [17]. A study published in 2015 suggested that patients with high-risk features for PM, such as T4 stage, mucinous tumors, Krukenberg tumors, or elevated CEA levels, could benefit from second-look laparoscopic surgery for early detection [11].

Adjuvant chemotherapy was associated with an increased risk of PM in our analysis, likely reflecting the advanced disease stage and poor prognosis of patients who received this treatment rather than the chemotherapy itself causing PM. This aligns with findings from a study reporting poor median overall survival in patients diagnosed with PM within a year of adjuvant chemotherapy administration [18].

This study has several limitations that must be acknowledged. Its retrospective design may introduce selection and information biases due to reliance on existing medical records, which might lack standardization or completeness. As a single-center study, the findings are limited in generalizability to other healthcare settings in Saudi Arabia or globally, where patient demographics, healthcare access, and treatment protocols may differ. Additionally, the small sample size for specific subgroups, such as those with signet cell histology or perforation, reduces statistical power to detect significant associations. The lack of molecular and genetic data limits insights into the role of specific biomarkers or genetic mutations in the development of PM. By excluding patients with radiological or intraoperative evidence of PM at the time of primary resection, the study may underrepresent the broader spectrum of PM. Moreover, despite multivariate analyses, unmeasured confounders such as lifestyle factors, comorbidities, or variations in adjuvant treatments may influence the results. Finally, the study does not assess the impact of different treatment strategies, such as CRS-HIPEC, on survival outcomes. Addressing these limitations in future multicenter, prospective studies with larger sample sizes and molecular data is essential to enhance the understanding of PM in CRC patients.

## Conclusions

Our study aims to report the incidence and demographics of patients presenting with PM and the predictive value of developing PM following primary CRC resection. We identified mucinous tumors and ovarian metastasis as significant risk factors for PM development. Early identification of these risks may help determine which patients would benefit from CRS-HIPEC. Overall, our findings provide valuable insights into the management and outcomes of patients with PM. Further research and clinical trials are warranted to explore novel therapeutic approaches and refine existing treatment protocols for improved outcomes in PM management.
